# The immediate effects of a single bout of aerobic exercise on oral glucose tolerance across the glucose tolerance continuum

**DOI:** 10.14814/phy2.12114

**Published:** 2014-08-28

**Authors:** Sine H. Knudsen, Kristian Karstoft, Bente K. Pedersen, Gerrit van Hall, Thomas P. J. Solomon

**Affiliations:** 1 Department of Infectious Diseases, The Centre of Inflammation and Metabolism and the Centre for Physical Activity Research, Rigshospitalet, University of Copenhagen, Copenhagen, Denmark; 2 Clinical Metabolomics Core Facility, Rigshospitalet, University of Copenhagen, Copenhagen, Denmark; 3 Department of Biomedical Sciences, Panum Institute, University of Copenhagen, Copenhagen, Denmark

**Keywords:** Glucose kinetics, oral glucose tolerance test, physical activity, type 2 diabetes

## Abstract

We investigated glucose tolerance and postprandial glucose fluxes immediately after a single bout of aerobic exercise in subjects representing the entire glucose tolerance continuum. Twenty‐four men with normal glucose tolerance (NGT), impaired glucose tolerance (IGT), or type 2 diabetes (T2D; age: 56 ± 1 years; body mass index: 27.8 ± 0.7 kg/m^2^, *P* > 0.05) underwent a 180‐min oral glucose tolerance test (OGTT) combined with constant intravenous infusion of [6,6‐^2^H_2_]glucose and ingestion of [U‐^13^C]glucose, following 1 h of exercise (50% of peak aerobic power) or rest. In both trials, plasma glucose concentrations and kinetics, insulin, C‐peptide, and glucagon were measured. Rates (mg kg^−1^ min^−1^) of glucose appearance from endogenous (*R*_aEndo_) and exogenous (oral glucose; *R*_a__OGTT_) sources, and glucose disappearance (*R*_d_) were determined. We found that exercise increased *R*_aEndo_, *R*_aOGTT_, and *R*_d_ (all *P* < 0.0001) in all groups with a tendency for a greater (~20%) peak *R*_aOGTT_ value in NGT subjects when compared to IGT and T2D subjects. Accordingly, following exercise, the plasma glucose concentration during the OGTT was increased in NGT subjects (*P* < 0.05), while unchanged in subjects with IGT and T2D. In conclusion, while a single bout of moderate‐intensity exercise increased the postprandial glucose response in NGT subjects, glucose tolerance following exercise was preserved in the two hyperglycemic groups. Thus, postprandial plasma glucose responses immediately following exercise are dependent on the underlying degree of glycemic control.

## Abstract (precis)

This study shows that following an exercise bout, plasma glucose concentrations during an oral glucose tolerance test are increased in subjects with normal glucose tolerance, but unchanged in subjects with impaired glucose tolerance or type 2 diabetes. While rates of glucose disappearance and rates of glucose appearance from endogenous sources and from orally ingested glucose were all increased following exercise, there was a 20% greater peak value for the rate of orally ingested glucose appearance in normal glucose tolerant subjects, when compared to IGT and T2D subjects. In summary, postprandial plasma glucose responses immediately following exercise are dependent on the underlying level of glycemic control.

## Introduction

Glycemic control is key in the management of type 2 diabetes (T2D), with postprandial glucose proposed as a better predictor of diabetes‐related complications than fasting blood glucose or HbA_1c_ (Cavalot et al. 2011). Therefore, studying postprandial glucose fluctuations has high physiological and clinical relevance. Aerobic exercise is prescribed clinically to prevent and treat T2D because it improves glycemic control (Church et al. 2010) and insulin sensitivity (Coker et al. 2009; Slentz et al. 2011) in obese and hyperglycemic individuals. However, exercise training is commonly accompanied by improvements in aerobic fitness and weight loss which independently influence glucose metabolism (Ivy 1997; Coker et al. 2009). A single bout of aerobic exercise, that does not alter fitness or body composition, is sufficient to increase insulin sensitivity both in healthy (Richter et al. 1989), prediabetic (Devlin and Horton 1985), and T2D subjects (Burstein et al. 1990; Bordenave et al. 2008). Nonetheless, the specific effect of a single exercise bout on postprandial glycemic control is far less consistent (Nazar et al. 1987; Rogers et al. 1988; Pestell et al. 1993; King et al. 1995; Larsen et al. 1997; Bonen et al. 1998; Baynard et al. 2005; Venables et al. 2007; van Dijk et al. 2013; Gonzalez et al. 2013; Roberts et al. 2013; Oberlin et al. 2014; Rynders et al. 2014) likely due to between‐study differences in the subject populations studied, the pre‐exercise nutritional state, the exercise modality, and/or the method and timing of the postprandial measure following exercise.

In healthy individuals, postprandial glucose tolerance has been shown to be increased, unchanged, or decreased in the hours after a single bout of aerobic exercise (Nazar et al. 1987; Pestell et al. 1993; King et al. 1995; Bonen et al. 1998; Rose et al. 2001; Roberts et al. 2013). In contrast, in prediabetic and T2D subjects postprandial glucose tolerance after a single bout of exercise has been found to be improved in some studies (Nazar et al. 1987; Oberlin et al. 2014; Rynders et al. 2014), while unchanged in others (Rogers et al. 1988; Larsen et al. 1997; Baynard et al. 2005; Venables et al. 2007), but a deterioration of oral glucose tolerance immediately following exercise has never been found. As such, it appears that the immediate effect of a single bout of exercise on postprandial plasma glucose levels differ between healthy and diabetic subpopulations suggesting that it may be dependent on the subject's underlying glycemic state. However, this has never been systematically investigated.

Determining exogenous and endogenous glucose flux in the postprandial period will add a mechanistic understanding of the above‐described observations. Therefore, in the current study we investigated the immediate effects of a single bout of moderate‐intensity aerobic exercise on glucose kinetics during an oral glucose tolerance test, in age‐ and body mass index (BMI)‐matched groups representing the entire glucose tolerance continuum: normal glucose tolerance (NGT), impaired glucose tolerance (IGT), and T2D. We hypothesized that the effect of a single bout of exercise on plasma glucose kinetics following oral glucose ingestion would be influenced by the subject's underlying level of oral glucose tolerance.

## Methods

### Subjects

Twenty‐four male subjects were recruited from the local area and were screened with a medical history and physical examination, blood chemistry analyses, and an oral glucose tolerance test (OGTT). Subjects were stratified as having NGT (*n *=**8), IGT (*n *=**8), or T2D (*n *=**8), based on WHO definitions (WHO & IDF Consultation 2006). Subjects were recruited with the intention to match the three groups for age and BMI. Data from NGT and T2D subjects – that are unrelated to the primary aim of this study – have been published previously (Knudsen et al. 2013). Individuals were included for participation if they were between 45 and 65 years old and had BMI between 25 and 35 kg/m^2^, and excluded from participation if they (1) were treated with insulin; (2) had unstable weight (>5 kg in previous 6 months); (3) had an illness that contraindicated physical activity; or (4) demonstrated any evidence of current or previous hematological, renal, hepatic, cardiovascular, or pulmonary disease. All enrolled subjects underwent a dual‐energy x‐ray absorptiometry (DXA) scan to determine whole‐body adiposity and fat‐free mass. All DXA images were analyzed by the same investigator using Encore 2004 software (GE Medical Systems Lunar Prodigy, Fairfield, CT). Subjects also performed an exercise test on a bicycle ergometer (Monark 839E; Monark, Varberg, Sweden) to determine maximal aerobic capacity (VO_2max_) with indirect calorimetry (Cosmed Quark b2, Rome, Italy) reflecting fitness level, as well as maximal power output (Watt [*W*]_max_) and heart rate (HR_max_) to calculate individual exercise workloads. Furthermore, subjects filled out a questionnaire reflecting habitual activity level modified from The Minnesota Leisure Time Physical Activity Questionnaire (Taylor et al. 1978). The study was approved by the Scientific Ethics Committee of the capital Region of Denmark (file no. H‐3‐2010‐127) in accordance with the Helsinki Declaration, and subjects gave oral and written consent to participate. Also, the study was registered on www.clinicaltrials.gov (NCT01607931).

### Design

On two occasions, separated by a minimum of 14 days, each subject underwent a 180‐min OGTT immediately following an hour of exercise (exercise trial) or rest (rest trial) in a randomized order. Exogenous and endogenous glucose kinetics were measured during the OGTT. Exercise was performed as 1 h of cycle ergometry at 50% of *W*_max_ and 60–90 rounds per minute (rpm). Power output was fixed during the entire trial. Heart rate was measured continuously and all trials were supervised by the same study investigator. For 3 days, prior to each trial, subjects were instructed by the same study investigator to complete diet records to ensure that daily energy intake (measured as the mean number of calories consumed per day during the 3 days prior to each trial) and daily macronutrient composition (measured as the mean percentage of energy derived from carbohydrate, fat or protein ingestion during the 3 days) were not different between trials. Diet records were analyzed by the same investigator using DanKost Sport 2000 software (Danish Catering Centre, Herlev, Denmark). During the three pretrial days, subjects were also instructed to refrain from physical activity and to pause any antidiabetic (metformin [*n *=**7], DPP4 inhibitors [*n *=**1], and sulfonylureas [*n *=**1]), antihypertensive (*n *=**3), or statin drugs (*n = *7).

### Experimental protocol

Each trial was performed after an overnight fast (~10 h). When arriving in the laboratory, catheters were placed in an antecubital vein in each arm – one for blood sampling and one for tracer infusion. Baseline blood samples were drawn at *T *=**−90 min and a primed (30 *μ*mol/kg multiplied by fasting plasma glucose/5), continuous (0.3 *μ*mol kg^−1^ min^−1^) infusion of [6,6‐^2^H_2_]glucose began. During the exercise trial, exercise was commenced at *T *=**−60 min. Immediately after rest/exercise at *T *=**0 min, a 180‐min OGTT was started by ingestion of a 300‐mL solution containing 73 g of anhydrous glucose and 2 g of [U‐^13^C]glucose. Sterility and pyrogen‐tested tracers were purchased from Cambridge Isotope Laboratories (Cambridge, MA) and prepared on the day of the test under aseptic conditions. Blood samples were collected at *T *=**−90, −60, −30, 0 min and every 10 min during OGTT for determination of plasma glucose and tracer enrichment, and every 30 min for the measurement of serum insulin, C‐peptide, and plasma glucagon. Venous blood was collected into the following tubes: heparin syringes for glucose analyses; Vacuettes (Becton‐Dickinson, Franklin Lakes, NJ) containing sodium fluoride for glucose enrichment analyses, Vacuettes containing EDTA and 10,000 kiU/mL aprotinin for glucagon analyses; and serum‐separation Vacuettes for insulin and C‐peptide analyses. Blood samples for plasma collection were immediately placed on ice and subsequently centrifuged (3500*g*, 15 min, 4°C), and the plasma was separated and stored at −80°C until analyses. Samples for serum collection were allowed to clot at room temperature for 30 min before centrifugation and subsequently sent for insulin and C‐peptide analysis at the Department of Clinical Biochemistry at Rigshospitalet.

### Measurements

Plasma glucose was measured by the glucose‐oxidase method (ABL 700; Radiometer, Brønshøj, Denmark). Serum insulin and C‐peptide were determined by electrochemiluminescence immunoassay (E‐Modular; Roche, Basel, Switzerland). Plasma glucagon concentrations were determined by radioimmunoassay (RIA; Millipore, MI). Plasma adrenaline and noradrenaline concentrations were determined by RIA (LND, Nordhorn, Germany). Plasma [6,6‐^2^H_2_]glucose and [U‐^13^C]glucose enrichments were quantified using liquid chromatography tandem mass spectrometry (API 3000 LC/MS/MS System; Applied Biosystems, Foster City, CA) using a hexobenzoyl derivatization method, as described previously (Oehlke et al. 1994).

### Calculations

Glucose, insulin, C‐peptide, and glucagon responses were calculated as area under the curve (AUC) during rest/exercise (*T *=**−90 to 0 min) and during OGTT (*T *=**0 to 180 min). The initial insulin and C‐peptide responses during the OGTT were determined as the incremental response during the first 15 min (Gerich 2002), calculated as delta of *T *=**0 and *T *=**15 (Δ0–15 min). Glucose kinetics were calculated using a nonsteady state single‐pool model as described previously (Steele 1959; Wolfe and Chinkes 2005). Total rate of glucose appearance (*R*_aTotal_) and disappearance (*R*_d_) were determined from plasma [6,6‐^2^H_2_]glucose enrichment. Rate of exogenous oral glucose appearance (*R*_aOGTT_) was determined from plasma [U‐^13^C]glucose enrichment (Proietto 1990). Rate of endogenous glucose appearance (*R*_aEndo_) was calculated as the difference between total *R*_a_ and *R*_aOGTT_. The postprandial suppression of *R*_aEndo_ was determined as the incremental response during the first 20 min, calculated as delta of *T *=**0 and *T *=**20 (Δ0–20 min). Glucose clearance during rest/exercise (*T *=**−90 to 0 min) and during OGTT (*T *=**0–180 min) was determined as *R*_d_ divided by plasma glucose.

### Statistics

One‐way analysis of variance (ANOVA) was used to compare between‐group baseline characteristics and AUC measures. Bonferroni post hoc tests were used to examine differences between group means. Two‐way repeated measures ANOVA was used to compare time and trial differences in total responses of plasma metabolites and glucose kinetics as well as pre‐/post‐exercise catecholamine levels within and between groups. Bonferroni post hoc tests were used to examine differences between trial means. Paired *t*‐tests were used to compare Δ and AUC values from control and exercise trial within groups. All data are presented as mean ± SEM. All analyses were conducted using Prism v4 (GraphPad Software, San Diego, CA) and statistical significance was accepted when *P* < 0.05.

## Results

### Subjects

Subject characteristics are presented in Table 1. Subjects with NGT, IGT, and T2D were not significantly different with respect to age and BMI; however, fat‐free mass was higher in NGT subjects compared to IGT (*P* = 0.05). Fasting glucose levels were highest in subjects with T2D (*P* < 0.05), and 2‐h OGTT glucose values were progressively higher across the groups (NGT < IGT < T2D, *P* < 0.05, −*P* < 0.0001). Furthermore, groups did not differ in habitual activity levels or VO_2max_ corrected for fat‐free mass.

**Table 1.** Subject characteristics.

	Overweight/Obese		
	NGT	IGT	T2D
*N*	8	8	8
Age (years)	53.6 ± 1.8	54.5 ± 2.6	59.9 ± 2.5
Weight (kg)	93.5 ± 5.3	88.1 ± 4.3	88.2 ± 2.1
BMI (kg/m^2^)	28.2 ± 1.6	27.2 ± 1.1	27.9 ± 1.0
Fat (%)	27.2 ± 3.2	31.0 ± 2.0	28.8 ± 2.1
Fat‐free mass (kg)	66.4 ± 2.0	59.4 ± 2.3^*^	60.7 ± 1.5
Fasting glucose (mmol/L)	5.5 ± 0.1	5.7 ± 0.2	8.1 ± 0.9^*^(^**^)
Fasting insulin (pmol/L)	5.2 ± 9.5	67.6 ± 16.0	72.1 ± 6.7
2 h OGTT glucose (mmol/L)	6.5 ± 0.3	10.3 ± 0.7^*^	14.6 ± 1.2^*^(^**^)
VO_2max_ (L/min)	3.526 ± 0.213	2.698 ± 0.158^#^	2.958 ± 0.319
VO_2max_ (mL/kg FFM per min)	53.0 ± 2.6	45.8 ± 3.0	48.2 ± 4.0
Habitual activity (kcal/day)	210.3 ± 62.7	249.5 ± 73.3	382.2 ± 72.9

NGT, normal glucose tolerance; IGT, impaired glucose tolerance; T2D, type 2 diabetes; BMI, body mass index; OGTT, oral glucose tolerance test; VO_2max_, maximal oxygen consumption during exhaustive incremental exercise. Data are presented as mean ± SEM. Group means were compared using one‐way ANOVA.

Statistically significant differences are indicated by *P < 0.05 vs. IGT and (**)*P* < 0.05 − 0.0001 vs. NGT. Statistically tendency is indicated by ^#^*P* = 0.07.

### Diet

Daily energy intake and dietary macronutrient composition is shown in Table 2. No significant differences were detected by ANOVA or *t*‐tests between any of the groups or between trials.

**Table 2.** Meal composition during three pretrial days.

	Overweight/Obese					
	NGT		IGT		T2D	
	Rest	Exercise	Rest	Exercise	Rest	Exercise
Energy intake (kcal/day)	2735 ± 422	2695 ± 270	2004 ± 236	1947 ± 241	2241 ± 200	2114 ± 262
CHO (%)	49.2 ± 3.0	49.0 ± 3.6	48.5 ± 5.1	48.6 ± 3.0	48.7 ± 3.1	45.1 ± 3.1
FAT (%)	28.1 ± 3.0	28.7 ± 2.8	35.6 ± 4.5	32.8 ± 2.5	32.8 ± 2.3	31.7 ± 2.2
PRO (%)	18.8 ± 1.5	18.8 ± 1.3	15.9 ± 1.0	20.0 ± 2.6	17.2 ± 1.3	18.4 ± 2.2

NGT, normal glucose tolerance; IGT, impaired glucose tolerance; T2D, type 2 diabetes; CHO, FAT, and PRO, calories of carbohydrate, fat, and protein ingested expressed as a percentage of the total energy intake. Data are presented as mean ± SEM of the 3 days prior to rest and exercise trials.

### Exercise

All subjects finished the 1 h of cycle ergometry exercise and exercise data are presented in Table 3. Exercise was performed at 49.7 ± 0.5% of *W*_max_ at 60–90 rpm with a mean heart rate of 116.5 ± 3.7 beats per minute (bpm) for all groups. Exercise intensity (% of *W*_max_) was not different between groups.

**Table 3.** Exercise data.

	Overweight/Obese			Mean
	NGT	IGT	T2D	
Mean work load (*W*)	128.9 ± 8.1	97.7 ± 12.2	107.1 ± 11.3	116.8 ± 7.7
Percentage of maximum work load (% *W*_max_)	50.1 ± 1.0	47.7 ± 1.2	49.4 ± 0.5	49.7 ± 0.5
Mean heart rate (bpm)	114.8 ± 5.2	114.5 ± 6.8	117.9 ± 5.0	116.5 ± 3.7

NGT, normal glucose tolerance; IGT, impaired glucose tolerance; T2D, type 2 diabetes; *W*, watt; bpm, beats per minute. Data are presented as mean ± SEM. Group means were compared using one‐way ANOVA.

### Plasma glucose

**Figure 1.**
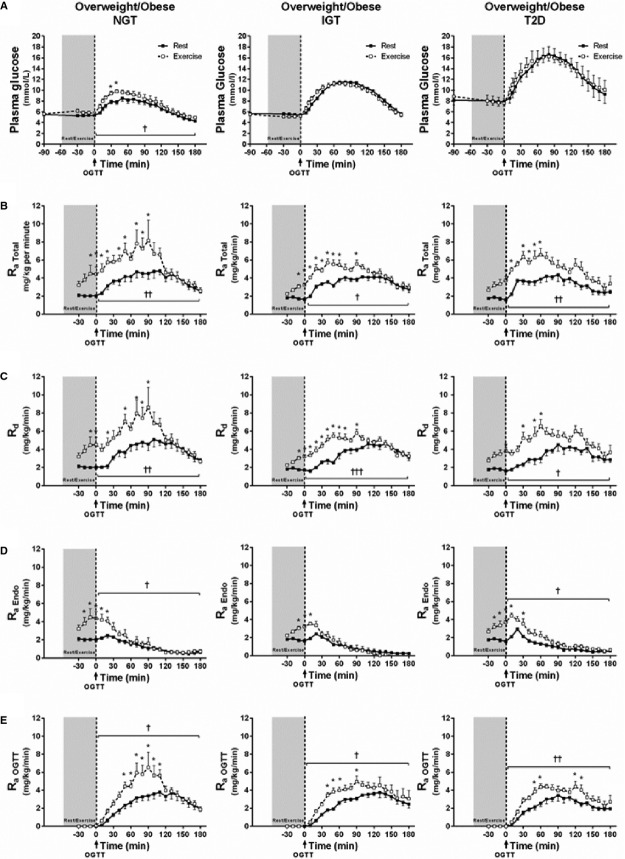
Glucose kinetics during rest/exercise and OGTT. Following an overnight fast, *n *=**24 subjects with normal glucose tolerance (NGT), impaired glucose tolerance (IGT), and type 2 diabetes (T2D) underwent an OGTT after a 1‐h period of rest or exercise. The data show rates of (A) plasma glucose, (B) total glucose appearance [*R*_aTotal_], glucose disappearance [*R*_d_], (C) endogenous glucose appearance [*R*_aEndo_], and (D) oral glucose appearance [*R*_a__OGTT_] during the rest (black squares; ■) and exercise (white squares; □) trials. Data are presented as mean ± SEM. Two‐way repeated measures ANOVA showed a significant effect of time and trial in NGT subjects for plasma glucose ([A]: *P* < 0.0001) and in all of the three groups for *R*_aTotal_ ([B]: *P* < 0.0001, all), *R*_d_ ([C]: *P* < 0.0001, all), *R*_aEndo_ ([D]: NGT,* P* < 0.0001; IGT,* P* < 0.01; and T2D, *P* < 0.0001), and *R*_a__OGTT_ ([E]: *P* < 0.0001, all). Bonferroni post hoc test revealed between‐trial differences (rest vs. exercise) indicated by *(*P* < 0.05–*P* < 0.001). Two‐way repeated measures ANOVA showed a significant time × trial interaction for *R*_aTotal_ ([B]: NGT,* P* < 0.001; IGT,* P* < 0.0001), *R*_d_ ([C]: NGT,* P* < 0.0001; IGT,* P* < 0.0001), *R*_aEndo_ ([D]: NGT; IGT; T2DM, *P* < 0.0001), and *R*_a__OGTT_ ([E]: NGT,* P* < 0.0001; IGT,* P* < 0.05; T2D, *P* = 0.09). Paired *t*‐tests showed that in the exercise trial during the OGTT (AUC) there was a significantly greater plasma glucose in NGT subjects ([A]: *P* < 0.05) and in all groups in *R*_aTotal_ ([B]: NGT,* P* < 0.01; IGT,* P* < 0.05; T2D, *P* < 0.01), *R*_d_ ([C]: NGT,* P* < 0.01; IGT,* P* < 0.001; T2D, *P* < 0.05), *R*_aEndo_ ([D]: NGT and T2D, both *P* < 0.05), and *R*_a__OGTT_ ([E]: NGT,* P* < 0.05; IGT,* P* < 0.05; T2D, *P* < 0.01), as indicated by ^†^(*P* < 0.05), ^††^(*P* < 0.01), and ^†††^(*P* < 0.001).

Fasting glucose values were not different between trials in any of the groups (Fig. 1A, *P* > 0.05). During exercise, plasma glucose (absolute values and AUC) was increased in the NGT group (Fig. 1A, *P* < 0.05 for both) while decreased in IGT subjects (Fig. 1A, *P* < 0.01 and *P* < 0.001, respectively). Furthermore, in subjects with T2D glucose levels tended to be lower immediately after exercise compared to baseline (*T *=**−90: 8.9 ± 1.1 vs. *T *=**0: 7.6 ± 0.9 mmol/L, *P* = 0.08). Two‐way repeated measures ANOVA revealed a main effect of time in all groups (*P* < 0.0001) and a main effect of trial in subjects with NGT (Fig. 1A, *P* < 0.0001). Post hoc analyses revealed that in NGT subjects, plasma glucose during OGTT was significantly higher in the exercise trial compared to the rest trial (Fig. 1A: *T *=**30 and *T *=**40 min, *P* < 0.01 and *P* < 0.05, respectively). Also, exercise increased the glucose response (AUC) to the OGTT in NGT subjects (Fig. 1A, *P* < 0.05); however, it was still lower than AUC glucose in subjects with T2D (*P* < 0.001). In contrast, the glucose response (AUC) to the OGTT in subjects with IGT and T2D was unaltered by exercise (Fig. 1A).

### Rate of glucose appearance (*R*_aTotal_)

No group differences in *R*_aTotal_ were found in the rest trial. Two‐way repeated measures ANOVA revealed an overall main effect of time and trial in subjects with NGT, IGT, and T2D (*P* < 0.0001, all), and a time × trial interaction in NGT and IGT groups (*P* < 0.001 and *P* < 0.0001, respectively). Post hoc analyses showed that *R*_aTotal_ was increased by exercise in all groups (Fig. 1B: NGT, *T *=**−10 to 50, 70 to 90 min; IGT, *T *=**−10 to 60, 90 min; T2D, *T *=**0 to 10, 30 to 60 min). Compared to the rest trial, *R*_aTotal_ (AUC) was increased during exercise in subjects with NGT, IGT, and T2D (*P* < 0.01, *P* < 0.0001, *P* < 0.01, respectively) as well as immediately after exercise (*T *=**−90 to 0 min, *P* < 0.01, all). Furthermore, *R*_aTotal_ (AUC) during OGTT was increased by exercise in subjects with NGT, IGT, and T2D (Fig. 1B, *P* < 0.01, *P* < 0.05 and *P* < 0.01, respectively).

### Rate of glucose disappearance (*R*_d_)

No group differences were found in the rest trial. Two‐way repeated measures ANOVA revealed an overall main effect of time and trial in subjects with NGT, IGT, and T2D (*P* < 0.0001, all), and a time × trial interaction in NGT and IGT groups (*P* < 0.0001, both). Post hoc analyses showed that *R*_d_ was increased by exercise in all groups (Fig. 1C: NGT, *T *=**10, 20, 40, 50, 70 to 90 min; IGT, *T *=**−10 to 60, 90 min; T2D, *T *=**10, 30, 50 min). Compared to the rest trial, *R*_d_ (AUC) was increased during exercise in subjects with NGT, IGT, and T2D (*P* < 0.05, *P* < 0.0001, *P* < 0.05, respectively) as well as immediately after exercise (*T *=**−90 to 0 min, *P* < 0.01, all). Furthermore, *R*_d_ (AUC) during OGTT was increased by exercise in subjects with NGT, IGT, and T2D (Fig. 1C, *P* < 0.01, *P* < 0.001, and *P* < 0.05, respectively).

### Rate of endogenous glucose appearance (*R*_aEndo_)

No baseline group differences were found in the rest trial; however, postprandial suppression (value below baseline) occurred at *T *=**40 min in NGT, *T *=**50 min in IGT, and *T *=**60 min in subjects with T2D. Moreover, postprandial suppression during the first 20 min (Δ0–20 min) was lower in subjects with T2D than NGT (*P* < 0.01). Two‐way repeated measures ANOVA revealed a main effect of time (*P* < 0.0001, all), trial (*P* < 0.0001, *P* < 0.01, and *P* < 0.0001, respectively), and a time × trial interaction in subjects with NGT, IGT, and T2D (*P* < 0.0001, all). Post hoc analyses revealed that *R*_aEndo_ was significantly higher in the exercise trial in all groups (Fig. 1D: NGT, *T *=**−20 to 20 min; IGT, *T *=**−10 to 10 min; T2D, *T *=**−10 to 10, 30 min). Compared to the rest trial, *R*_aEndo_ (AUC) was increased during exercise in subjects with NGT, IGT, and T2D (*P* < 0.01, *P* < 0.0001, *P* < 0.01, respectively). Also, *R*_aEndo_ (AUC) during OGTT was increased by exercise in subjects with NGT and T2D (Fig. 1D, *P* < 0.05, both).

### Rate of oral glucose appearance (*R*_aOGTT_)

No group differences were found in the rest trial. Two‐way repeated measures ANOVA revealed a main effect of time, trial (*P* < 0.0001, all) and time × trial interaction in all groups (*P* < 0.0001, *P* < 0.05, and *P* = 0.09, respectively). Post hoc analyses showed that *R*_aOGTT_ was significantly greater in the exercise trial in all groups (Fig. 1E: NGT, *T *=**50–100 min; IGT, *T *=**40–60, 90 min; T2D, *T *=**50, 60, 120, 130 min). Compared to the rest trial, *R*_aOGTT_ (AUC) during the OGTT was greater following exercise in all groups (Fig. 1E: NGT, *P* < 0.05; IGT, *P* < 0.05; T2D, *P* < 0.01), and although not statistically significant (*P* = 0.17), the peak *R*_aOGTT_ value following exercise was ~20% higher in NGT subjects compared to subjects with IGT or T2D.

### Rate of glucose clearance (*R*_d_/G)

**Figure 2.**
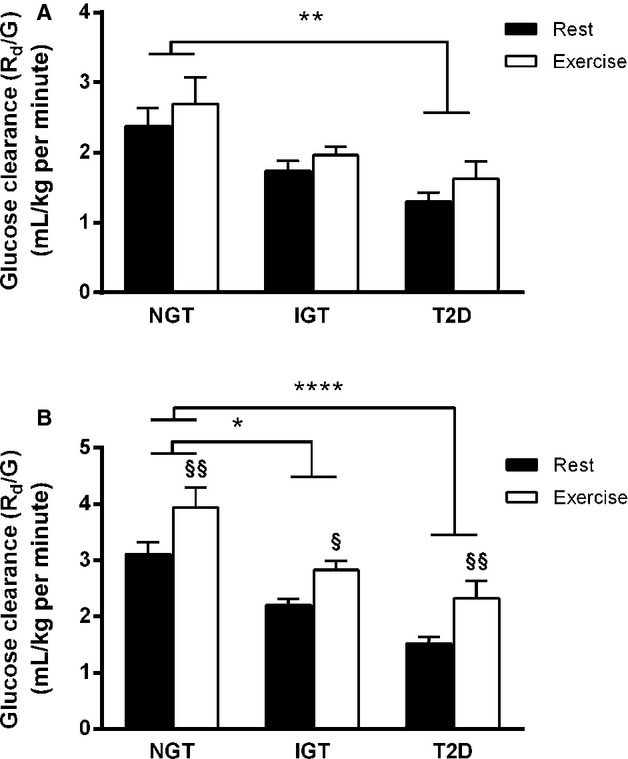
Glucose clearance during rest/exercise and OGTT. Following an overnight fast, *n *=**24 subjects with normal glucose tolerance (NGT), impaired glucose tolerance (IGT), and type 2 diabetes (T2D) underwent an OGTT after a 1‐h period of rest or exercise. The data show glucose clearance rates (*R*_d_/G) during (A) the 1‐h period of rest and exercise, and during (B) the OGTT, in the rest (black bars) and exercise (white bars) trials. Data are presented as mean ± SEM. (A) Two‐way ANOVA revealed a main effect of group (*P* < 0.01) and trial (*P* < 0.05) for glucose clearance during rest and exercise. Post hoc analysis revealed that compared to NGT subjects, glucose clearance was lower in subjects with T2D in both trials indicated as **(*P* < 0.01). (B) Two‐way ANOVA revealed a main effect of group (*P* < 0.001) and trial (*P* < 0.0001) for glucose clearance during OGTT. Post hoc analysis showed that compared to NGT subjects, glucose clearance was lower in subjects with IGT and T2D in both trials indicated as *(*P* < 0.05 and *P* < 0.0001, respectively). Post hoc analysis also showed that glucose clearance during OGTT was increased in all groups in the exercise trial compared to the rest trial indicated as §(B, NGT:* P* < 0.01, IGT:* P* < 0.05, and T2D: *P* < 0.01), and was still lower in subjects with IGT and T2D compared to NGT subjects (*P* < 0.05 and *P* < 0.01, respectively).

Two‐way ANOVA revealed a main effect of group (*P* < 0.01) and trial (*P* < 0.05) for glucose clearance during rest and exercise (Fig. 2A) indicating an increased clearance in all groups. Post hoc analysis revealed that during both rest and exercise glucose clearance was lower in subjects with T2D compared to NGT (Fig. 2A, both *P* < 0.01). Also, two‐way ANOVA revealed a main effect of group (*P* < 0.001) and trial (*P* < 0.0001) for glucose clearance during OGTT. Post hoc analysis showed that glucose clearance was lower in subjects with IGT and T2D in both trials when compared to NGT subjects (Fig. 2B, *P* < 0.05 and *P* < 0.0001, respectively). Also, post hoc analysis showed that glucose clearance during OGTT was increased in all groups in the exercise trial compared to the rest trial (Fig. 2B, NGT: *P* < 0.01, IGT: *P* < 0.05, and T2D: *P* < 0.01), and was still lower in subjects with IGT and T2D compared to NGT subjects (*P* < 0.05 and *P* < 0.01, respectively).

### Serum insulin

**Figure 3.**
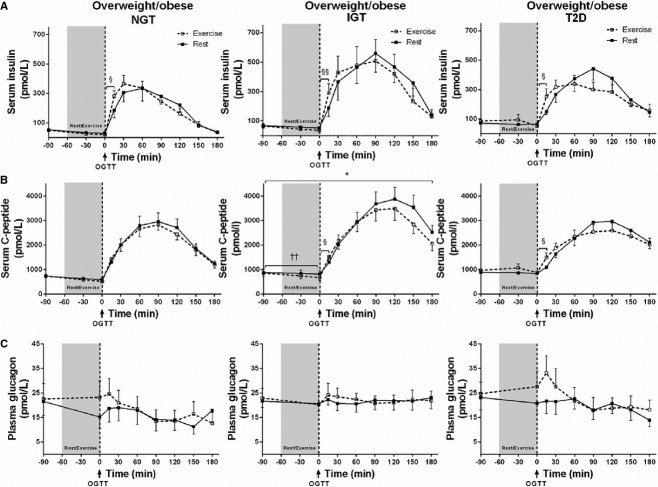
Metabolic responses during rest/exercise and OGTT. Following an overnight fast, *n *=**24 subjects with normal glucose tolerance (NGT), impaired glucose tolerance (IGT), and type 2 diabetes (T2D) underwent an OGTT after a 1‐h period of rest or exercise. The data show (A) serum insulin, (B) serum C‐peptide, and (C) plasma glucagon responses during the rest (black squares; ■) and exercise (white squares; □) trials. Data are presented as mean ± SEM. (A) The first‐phase incremental insulin response during OGTT (Δ0–15 min) was increased in all groups, indicated by §(NGT,* P* < 0.05; IGT,* P* < 0.01; T2D, *P* < 0.05). (B) Two‐way repeated measures ANOVA showed a significant main effect of trial for serum C‐peptide in the IGT group, indicated by *(*P* = 0.05). Also, paired *t*‐tests showed that first‐phase incremental C‐peptide response (Δ0–15 min) was increased in all groups, as shown by §(IGT,* P* < 0.01; T2D, *P* < 0.05). (C) No significant differences in glucagon were detected by ANOVA or *t*‐tests between any of the groups or between trials.

Fasting levels did not differ between any of the groups or between trials (Table 1, Fig. 3A, *P* > 0.05). Serum insulin (absolute values and AUC) was higher during exercise in subjects with T2D as compared to NGT (*P* < 0.05, both). In IGT subjects, the insulin response (AUC) to exercise showed a trend to be decreased as compared to rest (*P* = 0.058) with levels being lower immediately after exercise compared to baseline (85.8 ± 6.6 vs. 53.1 ± 5.9 pmol/L, *P* < 0.01). Two‐way repeated measures ANOVA revealed a main effect of time in all groups (*P* < 0.0001), but no main effect of trial (*P* > 0.05). No between‐group differences were found in the overall insulin response to the OGTT in the rest trial. Exercise did not affect the total insulin response (AUC) to the OGTT in any of the groups. However, the initial incremental insulin response (Δ0–15 min) to the OGTT was increased after exercise in all groups (Fig. 3A: NGT, *P* < 0.05; IGT, *P* < 0.01; T2D, *P* < 0.05).

### Serum C‐peptide

Fasting levels did not differ between any of the groups or between trials (Fig. 3B, *P* > 0.05). Initial incremental response (Δ0–15 min) to the OGTT was lower in subjects with T2D as compared to NGT (Δ269.8 ± 88.6 vs. Δ708.9 ± 141 pmol/L, respectively, *P* = 0.05). Also, serum C‐peptide (absolute values and AUC) was higher during exercise in subjects with T2D as compared to NGT (*P* < 0.05, both). In IGT subjects, the C‐peptide response (AUC) during exercise was decreased compared to the rest trial (Fig. 3B, *P* < 0.05). Two‐way repeated measures ANOVA revealed a main effect of time in all groups (*P* < 0.0001) and a main effect of trial in IGT subjects (Fig. 3B, *P* = 0.05), indicating that C‐peptide levels were lower in the exercise trial than in the rest trial. Post hoc analyses revealed no specific between‐trial differences. However, the first‐phase incremental C‐peptide response (Δ0–15 min) to the OGTT was increased in the exercise trial as compared to the rest trial in subjects with IGT and T2D (Fig. 3B, *P* < 0.01 and *P* < 0.05).

### Plasma glucagon

No significant differences in glucagon were detected between any of the groups or between trials (Fig. 3C).

### Plasma catecholamines

Despite a nonsignificant increase in plasma adrenaline following exercise in NGT subjects, no significant within‐ or between‐trial differences in either plasma adrenaline or noradrenaline were found (Table 4). Furthermore, catecholamine levels were not different between any of the groups.

**Table 4.** Catecholamines pre‐ and post exercise.

	Overweight/Obese					
	NGT		IGT		T2D	
	Pre	Post	Pre	Post	Pre	Post
Adrenaline (nmol/L)						
Rest	1.8 ± 0.4	1.7 ± 0.5	1.8 ± 0.4	1.8 ± 0.4	2.4 ± 0.6	2.5 ± 0.5
Exercise	2.2 ± 0.5	3.2 ± 1.2	2.2 ± 0.5	2.1 ± 0.3	1.7 ± 0.2	1.7 ± 0.3
Noradrenaline (nmol/L)						
Rest	18.8 ± 2.7	19.5 ± 2.0	10.3 ± 2.6	14.2 ± 3.6	15.8 ± 6.3	13.9 ± 3.7
Exercise	19.9 ± 2.9	21.5 ± 3.4	15.4 ± 3.6	17.9 ± 4.1	17.5 ± 4.4	18.9 ± 3.5

NGT, normal glucose tolerance; IGT, impaired glucose tolerance; T2D, type 2 diabetes. Data are presented as mean ± SEM. Two‐way repeated measures ANOVA was used to compare pre‐ and post differences in and between each group.

## Discussion

The main finding of our study was that while the postprandial plasma glucose concentration following an oral glucose load was increased immediately following a single bout of aerobic exercise in subjects with NGT, this effect on glucose tolerance following exercise was not observed in subjects with abnormal glycemic control (IGT and T2D). By systematically investigating groups representing the entire glucose tolerance continuum, for the first time these findings determine that the immediate effect of a single bout of aerobic exercise on oral glucose tolerance differs between healthy and diabetic subgroups, implying an impact of the underlying level of glycemic control.

The exercise‐induced increase in postprandial glucose response found in the present study is in accordance with previous findings (Nazar et al. 1987; Pestell et al. 1993; King et al. 1995) and could simply reflect normal postexercise glucose excursion in healthy subjects (Kjaer et al. 1986). Several factors may explain the lack of increase in postprandial oral glucose level found in IGT and T2D subjects in the present study. First, exercise‐induced elevation of plasma catecholamine levels (Kjaer et al. 1986, 1990) is known to increase hepatic glucose output in healthy subjects (Deibert and DeFronzo 1980; Sherwin and Sacca 1984), increasing glucose availability in the circulation. Even though we did not find significant increases in catecholamine levels, postprandial *R*_aEndo_ was increased by exercise in the present study. However, in contrast to Minuk et al. (1981) who showed that exercise failed to increase endogenous glucose production in T2D subjects, and in spite of a lower resting postprandial suppression of *R*_aEndo_ in our diabetic subjects, *R*_aEndo_ during OGTT was similar between groups following exercise. As such, differences in endogenous glucose production (which is predominantly hepatic) cannot explain the present group differences in exercise‐induced changes in oral glucose tolerance. Second, exercise increases muscle‐contraction‐induced glucose disposal via insulin‐independent GLUT‐4 translocation (Goodyear et al. 1990; Lund et al. 1995). Our results support previous findings that this exercise‐related mechanism is not impaired in subjects with poor glycemic control (Minuk et al. 1981; Martin et al. 1995; Dela et al. 1999) by showing that *R*_d_ during OGTT is not different between groups following exercise. That said, with our study design, *R*_d_ indeed reflects both insulin‐independent and insulin‐dependent glucose disposal. However, glucose clearance (a better indicator of the efficiency of glucose extraction from plasma than *R*_d_) was increased to the same extent by exercise in all three groups. This was seen when glucose clearance was normalized to either body mass (Fig. 2) or fat‐free mass (Data not shown but available from authors on request). This argues that group differences in exercise‐induced changes in postprandial plasma glucose levels are not dependent on group differences in muscle‐contraction‐induced glucose disposal. Third, prior work has shown that in healthy subjects a single bout of exercise can increase the appearance of orally ingested exogenous glucose in the circulation (Rose et al. 2001). In animal models, this phenomenon has been found to be related to the stimulatory effect of catecholamines (Ishikawa et al. 1997; Aschenbach et al. 2002). In our study, *R*_aOGTT_ following oral glucose ingestion was increased by exercise in all groups; however, this increase appeared to be greatest in NGT subjects. Following exercise *R*_aOGTT_ was increased more so during the earlier stage (0–120 min) of the OGTT in NGT subjects (ΔAUC 209.8 ± 52.0 mg kg^−1^ min^−1^) than in IGT (ΔAUC 139.8 ± 31.6 mg kg^−1^ min^−1^, *P* = 0.17) and T2D (ΔAUC 126.8 ± 25.2 mg kg^−1^ min^−1^, *P* = 0.29) subjects. These group differences were found along with a nonsignificant but ~20% greater peak value of *R*_aOGTT_ following exercise in NGT subjects (6.6 ± 1.7 mg kg^−1^ min^−1^) compared to IGT (5.0 ± 0.6 mg kg^−1^ min^−1^) and T2D (4.4 ± 0.3 mg kg^−1^ min^−1^) subjects. Despite being underpowered to detect these differences, in support of the findings of Rose et al. (2001), our data indicate that larger postexercise elevations in *R*_aOGTT_ in overweight/obese NGT subjects potentially explain the increment in the plasma glucose response during OGTT following exercise in that group, and the lack of response in IGT and T2D.

*β*‐adrenergic stimulation of the intestine by adrenaline increases glucose absorption in sheep and rats (Ishikawa et al. 1997; Aschenbach et al. 2002), potentially increasing orally ingested exogenous glucose appearance. Therefore, a diminished intestinal adrenaline effect following exercise in IGT and T2D subjects (Giacca et al. 1998) may lessen increments in *R*_aOGTT_. In our study, although plasma adrenaline levels increased following exercise in NGT but not IGT or T2D subjects, these observations were not statistically significant. From the present data, even though gastric emptying is most likely not affected at the exercise intensity used (van Nieuwenhoven et al. 1999), we also cannot rule out that differential gastric emptying between groups may also have influenced exogenous glucose appearance. Furthermore, it is important to note that the nonsignificant group differences in *R*_aOGTT_ may also be due to a caveat of our method in that exogenous glucose appearance estimated by tracer dilution of ingested [U‐^13^C]glucose does not account for possible group differences in the loss of glucose to splanchnic or hepatic uptake.

Interestingly, exercise increased the initial postprandial (∆0–15 min) responses of both serum C‐peptide and insulin in IGT and T2D subjects, indicating that exercise increases early‐phase glucose‐stimulated insulin secretion in hyperglycemic subjects. In contrast, in NGT subjects only initial postprandial insulin levels were increased, but not C‐peptide. In healthy subjects, it is known that exercise‐induced increases in adrenaline suppresses insulin secretion (Galbo et al. 1979; Minuk et al. 1981; Kjaer et al. 1986, 1990; Pestell et al. 1993); therefore, exercise‐induced increases in insulin secretion in IGT and T2D subjects could be due to lower sympathetic suppression of insulin secretion by adrenergic stimulation (Minuk et al. 1981; Krotkiewski and Gorski 1986). However, as mentioned above we did not find a significant difference in the adrenaline response to exercise between groups. As such, exercise‐induced changes in clearance of either insulin or C‐peptide may alternatively explain the differential changes in these variables between groups (Krotkiewski and Gorski 1986).

The increased initial insulin secretory (serum C‐peptide) response to the oral glucose load following exercise in subjects with IGT and T2D is an important observation. Diminution of first‐phase insulin secretion is one of the earliest detectable signs of *β*‐cell failure in the development of T2D (Gerich 2002); therefore, our results suggest that even a single bout of exercise may help restore postprandial *β*‐cell insulin secretory function. In spite of an increased first‐phase insulin secretion, overall insulin secretion was lowered in IGT subjects, while total postprandial insulin secretory response was unchanged in subjects with T2D. This suggests that the increased disappearance of glucose is due to enhanced insulin‐dependent and ‐independent glucose disposal rather than secretion of insulin and that this was sufficient to maintain glycemic control in IGT subjects.

The glucose‐lowering effect of exercise found in subjects with IGT and T2D (Minuk et al. 1981; Nazar et al. 1987; Martin et al. 1995; Kang et al. 1999; McClean et al. 2009) as compared to the increased plasma glucose levels during exercise and in the immediate postprandial period in NGT subjects (Nazar et al. 1987; Pestell et al. 1993; King et al. 1995), is in accordance with previous observations. However, the finding of an unaltered postprandial glucose level following exercise in IGT subjects is in contrast to the work of Nazar et al. (1987) who found decreased postprandial glucose levels immediately following exercise. Furthermore, Rynders et al. (2014) found that late‐phase glucose tolerance measured an hour after exercise cessation was intensity dependent. Higher intensity and/or longer duration of the exercise bout, probably eliciting a greater improvement in insulin dependent and/or independent glucose disposal, might explain this difference. Thus, the lower exercise intensity/duration and nonsignificant increases in catecholamine levels in our study may be the reason for an absence of improved glucose tolerance in subjects with IGT and T2D. However, parameters involved in glycemic control, such as insulin sensitivity and 24‐h glucose profile, have previously been shown to improve in both obese subjects with NGT and T2D by exercise of comparable duration and intensity (Bordenave et al. 2008; van Dijk et al. 2013; Newsom et al. 2013; Oberlin et al. 2014). For example, a single bout of exercise has been found to improve interstitial glucose levels that were continually measured over a 24‐h period in subjects with T2D (van Dijk et al. 2013; Oberlin et al. 2014). To our knowledge, our current study is the first to examine oral glucose tolerance immediately after exercise in T2D subjects. Thus, we hereby demonstrate for the first time that acute exercise‐induced increase in postprandial glucose level in NGT subjects is a phenomenon not seen in individuals with poor glycemic control. Furthermore, using evidence from studies showing improved glucose tolerance over a 24‐h postexercise period (van Dijk et al. 2013; Oberlin et al. 2014), it seems likely that this beneficial effect emerges beyond the time frame we have studied, that is, at least 2–3 h after the exercise bout, during the second and subsequent meals.

Prior knowledge of the effects of acute exercise on glucose kinetics in subjects with different underlying levels of glycemic control is compiled from several independent studies. The present study is the first to examine the effects of a single aerobic exercise bout on immediate glucose tolerance and postprandial glucose kinetics in age‐ and BMI‐matched groups of NGT, IGT, and T2D subjects simultaneously, representing the entire glucose tolerance continuum. Thereby, a strength of our study is that we can make group comparisons while directly controlling for differences in study designs and subject characteristics. However, our NGT subjects were in fact overweight/obese and since obesity per se is associated with impaired glucose tolerance (Pouliot et al. 1992) direct comparisons of the exercise‐induced changes in endocrine responses and glucose kinetics with prior studies that examined lean healthy NGT subjects should be made with caution. Absolute VO_2max_ (L/min) differed between NGT and IGT, and while not significant, the absolute VO_2max_ for T2D was substantially lower than NGT. This caused a ~30 watt difference in mean power output during exercise between NGT and IGT/T2D groups, which may have influenced our findings. However, this was not statistically different, and catecholamine levels were not different between groups also confirming that the exercise work load was similar between groups.

### Summary

Our study shows that while a single bout of aerobic exercise immediately increases the postprandial glucose response in NGT subjects, oral glucose tolerance following exercise is preserved in subjects with IGT and T2D. These data imply that the effect of a single bout of aerobic exercise on oral glucose tolerance is influenced by the underlying level of glycemic control. Future work should examine whether mechanisms of intestinal glucose absorption are influenced by the underlying level of glycemic control and consider the timing of postexercise feeding. This provides a future perspective in relation to designing exercise‐based treatments for diabetes‐related hyperglycemia.

## Acknowledgments

We express our gratitude to Lisbeth Andreasen (Department of Clinical Biochemistry, Rigshospitalet) for her technical assistance with clinical biochemistry assays. We also thank G. A. Wallis (University of Birmingham, UK) for providing intellectual critique on our manuscript.

## Conflict of Interest

None declared.
